# Microstructure Heterogeneity and Mechanical Properties of a High-Strength Ductile Laminated Steel by Electron Beam Welding

**DOI:** 10.3390/ma16083211

**Published:** 2023-04-19

**Authors:** Qiong He, Mingsai Wang, Bo Yang, Fengjiao Guo, Hao Ran, Wei Wei, Chao Zhang, Yu Zhai, Qingyuan Wang, Wenquan Cao, Chongxiang Huang

**Affiliations:** 1 School of Aeronautics and Astronautics, Sichuan University, Chengdu 610065, China; 2 Key Laboratory of Deep Earth Science and Engineering, Ministry of Education, Sichuan University, Chengdu 610065, China; 3 Central Iron and Steel Research Institute (CISRI), Beijing 100081, China

**Keywords:** high-strength steel, microstructure heterogeneity, TRIP effect, synergistic constraint, Lüders bands

## Abstract

The aim of this study is to fabricate high-strength steel with exceptional yield strength and superior ductility by employing a novel design approach of nanolamellar/equiaxial crystal “sandwich” heterostructures, utilizing rolling and electron-beam-welding techniques. The microstructural heterogeneity of the steel is manifested in the phase content and grain size, ranging from nanolamellae comprising a small quantity of martensite on both sides to the completely coarse austenite in the center, which are interconnected via gradient interfaces. The structural heterogeneity and phase-transformation-induced plasticity (TIRP) offer remarkable strength and ductility for the samples. Furthermore, the synergistic confinement of the heterogeneous structures leads to the formation of Lüders bands, which exhibit stable propagation under the TIRP effect and impede the onset of plastic instability, ultimately resulting in a significant improvement in the ductility of the high-strength steel.

## 1. Introduction

Metallic materials have played a pivotal role in shaping modern civilization. However, as the demand for development increases, so do the requirements for the strength and ductility of materials. The nano-grained or ultrafine-grained structures formed by severe plastic deformation can exhibit high strength but with a large loss of plasticity [1]. Therefore, achieving synergy between strength and ductility in materials is a challenging task and remains a major limitation in engineering applications. Fortunately, numerous studies [2,3,4,5] have shown that this problem can be addressed through material or structural design. One promising solution for achieving strength–ductility synergy is the use of heterostructures (HSs) [6], which include gradient structures [7], harmonic structures [8], bimodal and multimodal structures [9,10], etc. These structures are characterized by significant microstructural heterogeneities that comprise domains with varying strengths and sharp or gradient interfaces between them [11,12,13]. The resulting macroscopic strength heterogeneity and mechanical incompatibility between layers [14,15] generate strain gradients and changes in stress states, leading to synergistic strengthening and strain hardening. This effect enhances both strength and ductility [16,17,18], making heterostructures a universal strategy for realizing the strength–ductility synergy in metallic materials.

The TRIP effect, i.e., transformation-induced plasticity, is one of the most effective approaches to increase both strain hardening and ductility for steel. This effect is commonly observed in duplex/multiphase steels [19,20], TRIP steels [21], and bainitic steels [22] and is mainly manifested by the phase transformation during deformation to form the martensitic phase. It achieves a high strength and strain-hardening rate by introducing strain gradient at phase boundaries and delaying strain localization formation [23,24], in which the increase in strain-hardening rate benefits the ductility of steels. Hence, the TRIP effect is one of the effective means to ensure strength–ductility synergy for steels. Considering the positive effects of heterostructure and TRIP effect on mechanical properties, it is expected that the combination of the two can further improve the strength–ductility of steels. Recently, it has been reported that heterostructures can effectively promote the sustainable and stable development of martensitic transformation by synergistic constraints between mechanically incompatible layers [12,25,26]. Therefore, a heterogeneous structure combined with the TRIP effect is expected to produce an excellent combination of strength and ductility.

Distinct Lüders-like deformation has also been found in steels having austenite matrix or retained austenite that have been claimed to achieve both high strength and large elongation [27,28,29]. In many advanced high-strength steels, the Lüders elongation usually accounts for more than half of the total elongation [30,31]. For example, the deformed and partitioned steel (D&P steel) prepared by Huang et al. [30] achieved a strength of ~2.2 GPa and a ductility of ~16.5%, with a Lüders elongation of ~9%. So, Lüders-like deformation is key for the excellent mechanical properties of these steels. Meanwhile, it has been found that Lüders bands deformation and martensitic transformation affect each other. Martensitic transformation occurs during Lüders deformation and provides necessary strain hardening for suppressing local plastic instability [32,33]. However, the effect of HS on Lüders bands is not clear.

This paper combines the structural heterogeneity-induced synergistic effect, TRIP effect, and Lüders-like deformation in high-strength steel to optimize the strength–ductility synergy. The synergistic constraint by structural heterogeneity and the extra strain hardening by the TRIP effect may provide the necessary conditions for the sustained and stable propagation of Lüders-like deformation. The effect of structural heterogeneity on Lüders-like deformation is proposed for the first time. This strategy may be helpful to explore better tensile properties and deformation mechanisms of steels.

## 2. Experimental Methods

**Figure 1:**
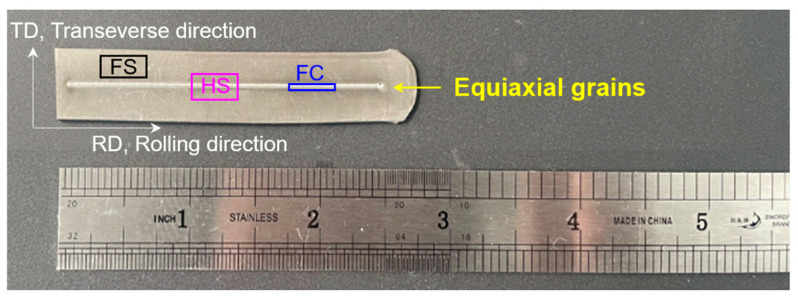
The plate after the welding treatment is completed, where HS, FC and FS stand for heterogeneous structure, freestanding center coarse and freestanding surface, respectively.

The chemical composition of the received high-strength steel plates (5 mm thick) is shown in Table 1. The as-received sample was homogenized at 1100 °C for 2 h and quenched in water. The homogenized plates were ground and polished, reducing the plate thickness to 2.5 mm. Additionally, the plates were rolled from 2.5 mm to 0.5 mm at 300 °C with a holding time of 3 min to obtain a nanoscale martensitic–austenitic dual-phase structure. Electron beam welding (EBW) was used to obtain equiaxial-grained austenite structure with a beam voltage of 60 kV, a current density of 10 mA, and a welding speed of 2000 mm/min in the rolling direction. The rolled plate is first ground and polished before EBW. Then, the center part of the rolled plate is bombarded with electron beam to make the grains grow back to form coarse crystalline austenite phase. The final plate is shown in Figure 1. The position marked by the yellow arrow in the middle of the plate is the equiaxed austenitic crystal structure formed by welding.

The microstructures of HS samples were observed by optical microscopy (OM) and transmission electron microscopy (TEM) using an FEI Tecnai G2 F20 field emission microscope. The fracture geometry and morphology across the fracture interface after tensile test were examined by scanning electron microscopy (SEM). The phase distribution and transition during tension were studied by X-ray diffraction (XRD), using CoKα radiation, in a scanning range of 40–110° at 0.02° s^−1^. To analyze the phase content, the raw XRD data were processed by Rietveld refinement using the TOPAS tool. At least three samples were used for phase content testing and calculations. The relevant data were statistically processed to obtain the average value and standard deviation.

**Figure 2:**
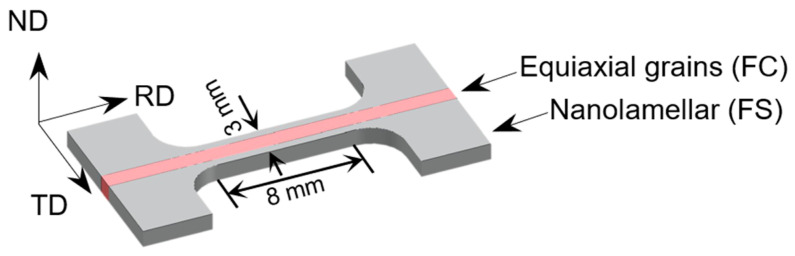
Schematic diagram of the tensile sample after welding is completed.

Dog-bone-shaped tensile specimens with a gauge length of 8 mm were machined parallel to the processing direction in EBW processed plate using a wire cutting machine. The cross-sectional dimensions of HS, freestanding center coarse (FC), and freestanding surface (FS) samples were 3 × 0.5 mm^2^, 1 × 0.5 mm^2^, and 2 × 0.5 mm^2^, respectively. HS, FC, and FS were taken from the positions shown in Figure 1. Figure 2 shows the schematic diagram of the HS stretching sample, where RD is the rolling direction, TD is the transverse direction, and ND is the normal direction. The equiaxial-grained center occupies ~32% along the scale-width direction. Uniaxial tensile tests were performed at room temperature (RT) at a strain rate of 5 × 10^−4^ s^−1^. To ensure reproducibility, a minimum of three samples of each sample type were used for tensile testing.

Vickers microhardness measurements were conducted before and after tension with a load of 100 gf for 15 s. Four independent positions were measured for each type of cross-section. The average value and standard deviation of these hardness values were taken. In situ 2D DIC was performed on the surface of HS sample with a short-focus optical lens. Random patterns were prepared by spraying black paints on white background before DIC imaging. The height contours on the surface of the HS samples after tensile test was measured by a Bruker Contour-I white light interferometer in a vertical scanning mode with a height resolution in depth of 20 nm.

## 3. Results

### 3.1. Nanolamellar/Equiaxial Grains “Sandwich” Heterostructures

**Figure 3:**
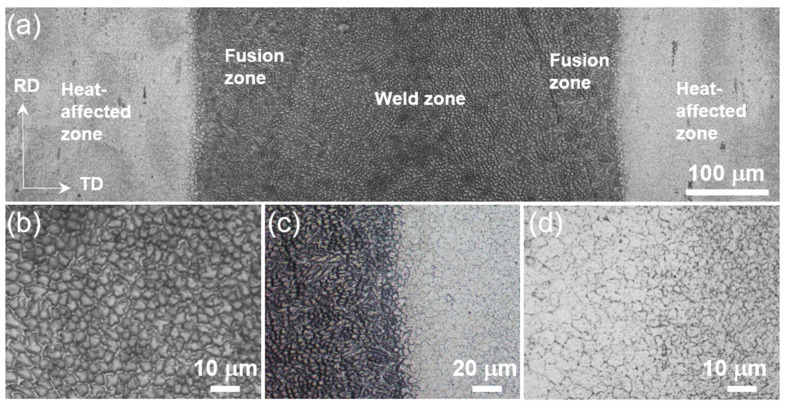
OM diagram showing the microstructure of the HS sample. (**a**) Overall shape of the core weld area; (**b**) shape of the middle weld area; (**c**) shape of the fusion zone at the boundary with the heat affected zone; and (**d**) shape of the heat affected zone.

**Figure 4:**
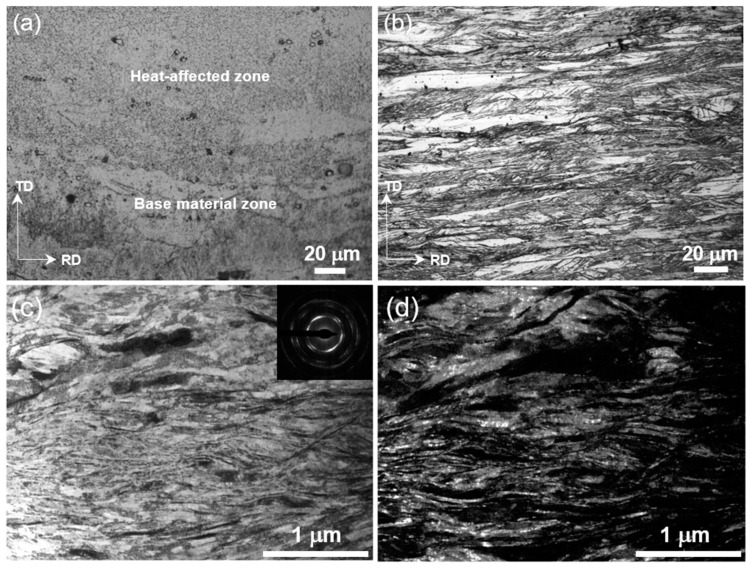
Microstructure of side layers in HS sample. (**a**) OM image at the boundary between the heat-affected zone and the base material zone; (**b**) OM image of the base material zone; and (**c**,**d**) TEM images of the base material zone.

Figure 3 shows the microstructure of the welding region, which mainly includes the weld zone, fusion zone, and heat-affected zone. The weld zone exhibits an obvious cell-like structure (Figure 3b), while the fusion zone shows a mixed microstructure of cell- and branch-like shapes (Figure 3c). The heat-affected zone has obvious grain growth with an equiaxed crystal structure (Figure 3d), and the grain size gradually decreases with increasing distance from the welding region. The microstructure of the sample approaches the lamellar structure away from the central welding region at ~450 µm. Figure 4a shows the structural morphology of the heat-affected zone and the base material zone, where the base material exhibits a more complex lamellar structure. Detailed OM observation on the matrix verifies the lamellar structure (Figure 4b). This lamellar structure is consistent with the rolled state sample that exhibits a dual-phase nanolamellar structure (Figure 4c). Thus, electron beam welding results in a heterogeneous structure that is manifested by equiaxed grains in the core and nanolamellar grains on both sides.

**Figure 5:**
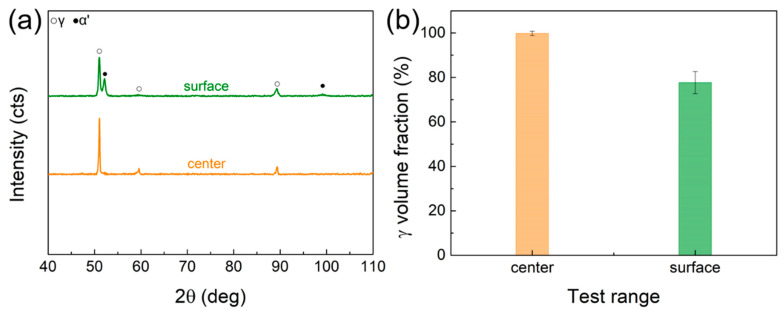
Phase composition of the core and side layers of HS sample. (**a**) XRD patterns for the center and surface layer; (**b**) austenite content in the center and surface layer.

The equiaxial grains in the core are composed of a full austenite phase, as determined by the XRD test (Figure 5). Figure 5b shows the average value of austenite content. The difference between the core and side layers of the “sandwich” HS sample can be characterized by 2 aspects: (1) the core has larger equiaxed grains, while it is nanolamellar layers on both sides; (2) the core is full of austenitic phase, while there is 20% martensitic phase of side layers. Such unique nanolamellar/equiaxial “sandwich” heterogeneous structures differ from conventional HSs composed of only grain size heterogeneity or phase content [18,34,35].

### 3.2. Tensile Mechanical Response

**Figure 6:**
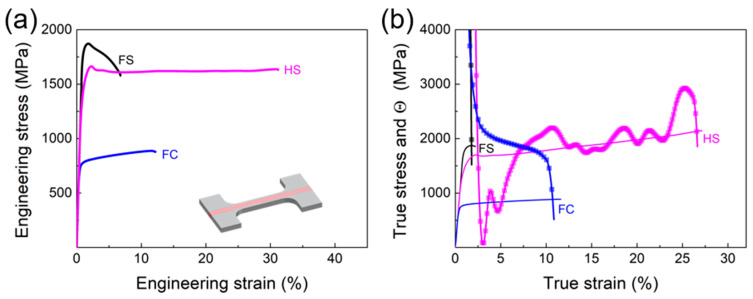
Tensile mechanical behaviors of HS, FC, and FS samples. (**a**) Engineering stress–strain curves. The insert in (**a**) is dog-bone-shaped tensile HS specimen, where the pink area is the weld. (**b**) True stress–strain and strain hardening (Θ) behaviors.

Figure 6a shows the static uniaxial tensile tests of the HS, FC, and FS samples. The FS sample exhibits ultra-high strength but very low ductility, while the FC sample has low strength and some ductility. The yield strength of the HS sample is twice as much as that of the FC sample and even close to that of the FS sample. This indicates that the coarse austenite structure at the center does not significantly reduce the strength of the HS sample. On the other hand, the elongation at fracture of the HS sample is significantly superior to that of the FS sample. Interestingly, the fracture elongation of the HS sample is also much higher than that of the FC sample. This suggests that the nanolamellar structure in side layers also has affected the ductility of the core coarse grains during the tension process. It is under the effect of heterogeneous structure that the HS samples can show excellent strength–ductility.

It is also observed in Figure 6b that for the HS sample, there is an obvious phenomenon of strain-hardening rate turn-up, i.e., extra hardening occurs during the tensile deformation. This phenomenon is not present in the FC and FS samples, suggesting that it is caused by the heterogeneous structure. Moreover, the stress–strain curve of the HS sample has upper and lower yield points, and the stress remains constant at lower yield stress with increasing strain afterwards. This indicates that Lüders bands deformation [27,36] might take place during tension.

**Figure 7:**
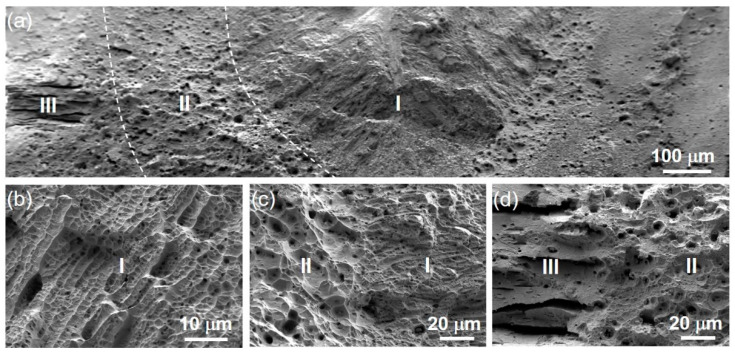
SEM micrographs of the fracture surface for HS sample. (**a**) Low-magnification image showing an overview fracture surface. (**b**–**d**) High-magnification images showing specific morphologies.

The fracture morphology of the HS specimen is shown in Figure 7. The fracture surface has three regions corresponding to the weld (Figure 3 and Figure 4): weld zone (I), heat affected zone (II) and matrix zone (nanolamellar structure zone) (III). The high magnification images in Figure 7b–d reveal that each region consisted of tough nests, with the columnar arrangement in Region I and interlaminar fractures parallel to the rolling surface in Region III, both of which are very ductile fracture patterns. Moreover, the fracture transition between regions is natural, and no tearing is observed, indicating synergistic deformation of three regions during tension.

### 3.3. Microstructural Evolution

**Figure 8:**
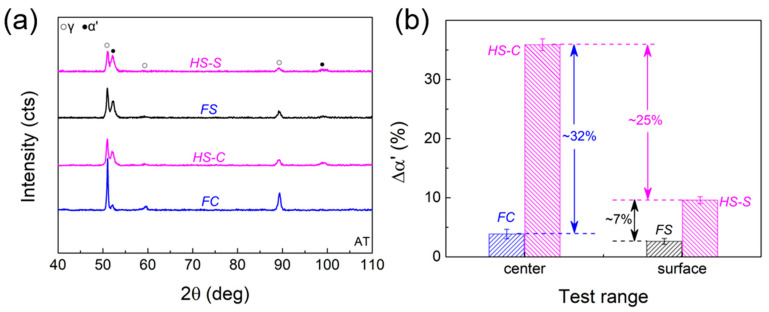
Phase content in different samples before and after tension. (**a**) XRD pattern of the sample after tensile deformation; (**b**) change in martensite content of the sample before and after tensile deformation.

The HS sample has a central layer with coarse-grained austenite (HS-C) and two side layers with a dual-phase nanolamellar structure (HS-S). Figure 8a presents the XRD patterns of the HS-C, HS-S, FC, and FS samples after tension. Figure 8b shows the average value of the martensite content. It indicates that the martensite content in the free core and side layer changes very little after the tension, indicating no martensitic phase transformation. For the HS sample, a large variation in martensite content is observed. The martensite increment in HS-C and HS-S layers is much greater than that in FC and FS samples. This suggests that the coarse-grained core and nanolamellar side layers experience intense interaction during tensile deformation to promote austenite-to-martensite transformation.

**Figure 9:**
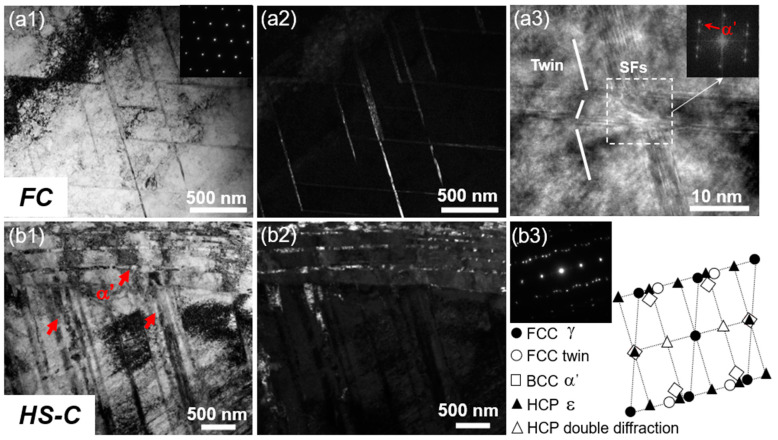
TEM microstructures of tensile samples. (**a1**,**a2**) Microstructures of freestanding center (FC) samples after tensile deformation; (**a3**) high-resolution TEM image showing the martensite in FC sample after deformation; (**b1**,**b2**) microstructures of HS sample after tensile testing; and (**b3**) diffraction pattern of (**b1**). The red arrows in (**a3**,**b1**) indicate the α′-martensite.

TEM observations after tensile tests show that the FS sample and HS-S layer have a similar microstructure as the initial nanolamellar structure. The difference is that the coarse-grained core underwent significant inconsistent change under the heterogeneous structure. Compared with weak deformation twinning and α′-martensite formed in the FC sample (Figure 9a1–a3), significant γ-austenitic phase, ε-martensitic phase, and α′-martensitic phase (Figure 9b1–b3) inside the HS sample are detected, indicating significant martensitic phase transformation during tensile deformation. There are obvious differences in microstructures of FC and HS-C samples after deformation, which is consistent with a significant difference in martensite content between them (Figure 8b). It is also illustrated that the coarse-grained core underwent more plastic deformation and martensitic phase transformation under the constraint of nanolamellar structure on both sides.

**Figure 10:**
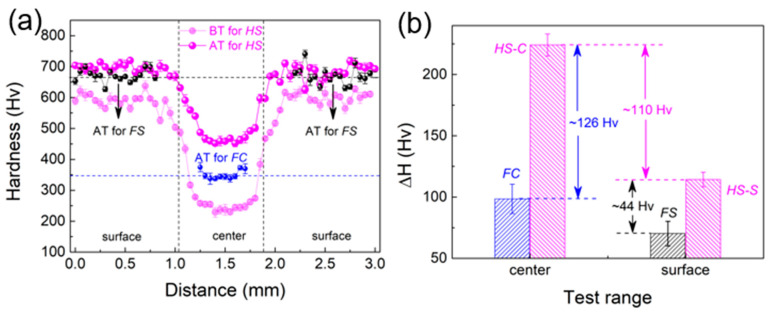
(**a**) Hardness distribution of FC, FS, and HS samples after tensile deformation along the scale width direction; (**b**) hardness difference before and after tension within the central core and surface layers.

The hardness distributions of FC, FS, and HS samples along the scale-width direction after tension (AT) is shown in Figure 10a, in which the light purple color indicates the hardness distribution of each sample before tension (BT). Calculation of the hardness difference before and after tension reveals that the incremental hardness in the core and side layers (HS-C and HS-S) is much larger than that of their corresponding freestanding layers (FC and FS), as shown in Figure 10b. The change in hardness increment is compatible with the change in phase content and microstructure (Figure 8 and Figure 9). Of greater interest is the fact that the core and side layers show significant additional hardening due to the heterostructure effect, which is reflected in the fact that the introduction of the coarse-grained core layer does not substantially reduce the strength of the sample (Figure 6a).

### 3.4. Strain Distribution

**Figure 11:**
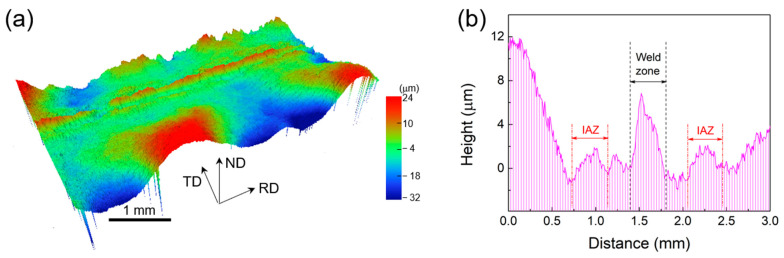
(**a**) 3D height contour measured on the surface of HS sample after tensile testing. (**b**) The distribution of statistical average height as a function of sample gauge width.

Figure 11a shows the 3D height contour diagram of a uniformly spaced section on the HS sample surface after tension. The red regions indicate higher heights than the blue ones. The center layer of the testing surface is higher than the side layers. Moreover, the side layers also exhibit some height increase, forming folds that intersect with the center. The height values in Figure 11a are measured along the RD direction and averaged along the TD direction, as shown in Figure 11b. It shows that: (1) there is a height difference between the two ends of the TD direction, corresponding to the fold-like shape on both sides in Figure 11a. Combined with Figure 6a, which shows the Lüders band during tension, this strip-like fold with varying heights is identified as the Lüders band, indicating (2) there is an abnormal elevation at the interface between the central layer and two side layers. This interface with high elevation variation is termed an interface affected zone (IAZ) [37].

**Figure 12:**
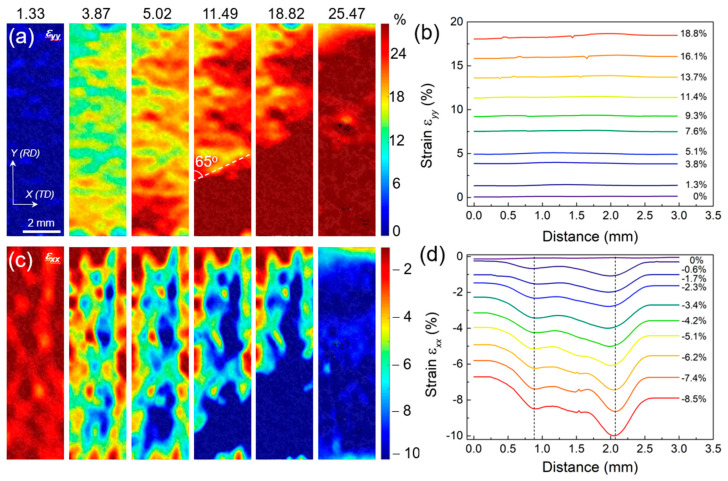
(**a**,**c**) are the contours of strain *ε_yy_* and *ε_xx_* mapped at different tensile strains, respectively. (**b**,**d**) are the distribution of statistical average strain *ε_yy_* and *ε_xx_* plotted as a function of distance from the width, respectively. The dotted lines mark the position of layer boundaries.

Figure 12 shows the strain distribution on the sample surface during tensile deformation, characterized by optical DIC. *ε_yy_* is the positive tensile strain along the tensile direction, and *ε_xx_* is the positive shrinkage strain along the sample width direction. Figure 12a,b show that *ε_yy_* does not differ significantly between the center layer and two side layers. Figure 12b shows that the tensile positive strain does not change depending on the position of the scale width in which it is located. This indicates that the deformation of the center layer and two side layers are synchronous and uniform in the tensile direction, and no delamination occurs [11]. The core and both sides deform uniformly and synchronously in the tensile direction. Lüders bands, i.e., the strain concentration band [38], are also visible in Figure 12a and expand with increasing tensile strain until they cover the whole sample. The distribution of *ε_xx_* is completely different from that of *ε_yy_*. Figure 12c shows that *ε_xx_* in the coarse-grained core layer is much higher than that in the nanolamellar layer on both sides. In Figure 12d, it is revealed an obvious interfacial gradient at the junction of the core layer and nanolamellar side layer, indicating a high strain gradient in the IAZ [11,35,38].

The 3D height profile and strain distribution of the sample can corroborate each other to obtain: (1) the heterogeneous structure of the HS sample produces Lüders bands on the surface during the deformation process, and the Lüders bands gradually extend to the whole sample with increasing strain; (2) the center layer and two side layers are deformed uniformly in the tensile direction simultaneously, and the tensile positive strain remains the same; (3) During the tensile deformation, the center layer is the first to deform plastically than the two side layers. Additionally, the positive shrinkage strain is transferred from the center to both sides, which affects both sides; (4) there is an interface influence zone at the junction between the center layer and two side layers, which shows a more obvious strain concentration and a peak under the positive shrinkage strain. Additionally, this interfacial influence zone exhibits an interfacial strain gradient.

## 4. Discussion

### 4.1. Extra Hardening and Strengthening

Using the rule of mixture (ROM) [39] to calculate the yield strength of the nanolamellar layers on both sides and the coarse-grained layer in the center, the calculation is given by the following:

$$\sigma_{ys}=\sum V_{i}\sigma_{i,\;ys}^{\prime },$$

where $V_{i}$ is the volume fraction of component *i*, and $\sigma_{i,\;ys}^{\prime }$ is the yield stress of component *i* when it is tensile deformed alone. The yield strength of the HS sample is calculated to be about 1452 MPa. On the other hand, experimental measurement shows a yield strength of about 1608 MPa, which is higher than the calculated value. This proves that extra strengthening is generated within the HS sample. This extra hardening and strengthening are attributed to two aspects: hetero-deformation-induced (HDI) hardening from the interaction of the heterogeneous layers [11,17,18], and the heterogeneous-structure-induced obvious TRIP effect that brings extra strengthening and hardening.

For the HS sample, the core coarse crystalline layer and the nanosheet layers on both sides are significantly different in terms of grain size, tissue structure, and phase content. As a result, there is inevitably a non-synergistic deformation phenomenon during the tensile deformation of the core coarse crystalline layer and the nanosheet layers on both sides. As shown in Figure 12, although the strain state of HS samples remains the same in the tensile direction, there is a significant difference in the transverse shrinkage direction. This difference is strong evidence of the incoherent deformation between the layers of HS samples. In order to adjust this incoherent deformation, it is necessary to accumulate strain gradients in the interlayer interface region to ensure the deformation continuity of samples. In addition, for the interfacial influence zone, Ma et al. [40] found that there is a significant geometrically necessary dislocation (GND) plugging near the coarse crystal/nanocrystal interface. This result suggests that the interlayer deformation inhomogeneity is achieved in the interface-influenced region by strain gradients and GND plugging. Meanwhile, the accumulation of GNDs in the strain gradient region leads to the development of long-range internal stresses, which can promote both strengthening and strain hardening, i.e., synergistic strengthening and strain hardening.

Furthermore, this synergistic strengthening, in turn, helps to promote phase transformation and thus enhance strain hardening. As seen in Figure 8, it is under the influence of the interaction between the middle layer and the sides that induce the formation of more martensitic phases. The martensitic phase, as a hard phase, shows a higher strength than the austenitic phase, which, to some extent, also contributes to the assurance of high strength of the HS samples. Additionally, the martensite formation process represents the generation of the TRIP effect, which provides an extra hardening effect. As a result, the HS samples show increased strain-hardening rate (Figure 6b) and extra hardness contribution (Figure 10) under the combined effect of structural heterogeneity and the TRIP effect.

### 4.2. Synergistic Deformation under Heterogeneous Structure

It is shown that the Lüders band persists throughout the tensile deformation of the HS sample (Figure 11 and Figure 12). The strain distribution under the Lüders band and the heterogeneous structure are interlaced. At the same time, obvious phase transformation is promoted (Figure 8). Therefore, analyzing the formation and propagation of the Lüders band strain can help understand how the heterogeneous structure and TRIP effect affect the strength and ductility of steel. The main interaction mechanisms between the nanolamellar side layer and the equiaxial-grained core layer in the HS sample during tensile deformation include: (i) the hard side layer constrains the plastic deformation of the soft core layer; (ii) the soft layer inhibits the necking fracture of the hard layer [41,42], and (iii) the geometrically necessary dislocation pile-up and HDI stresses at the interface form between the soft and hard layers. These mechanisms cause stress concentration at interfaces for the nucleation of deformation bands/Lüders bands (Figure 12c).

The heterogeneous structure affects both the formation and the stabilization of the Lüders band deformation. However, this plastic instability (Lüders band) is very easily destabilized and fractured in the region with stress concentration rather than continuous strain propagation. For example, the same upper and lower yield points appear in the FS sample (Figure 6a), but the sample destabilizes and fractures directly after the appearance of the yield drop, and no stable propagation of plastic instability is achieved. In the HS sample, the presence of heterogeneous structure improves the stability of Lüders deformation. According to Mohr’s circle of stress [43,44], elliptic instability has been reached for normal and shear stresses in the independent free surface layer but not yet for the heterogeneous structured surface layer. This means that under the same stress condition when internal strain concentration occurs, the surface layer can still deform stably under the aid of a heterogeneous structure. This influence is due to an extra constraint effect between the coarse-grained core layer and nanolamellar side layers. As a result, the heterostructure can ensure stable propagation of the Lüders band.

Of course, the Lüders band propagation is influenced by the martensitic phase transformation in addition to heterostructure. The heterostructure changes the stress distribution inside the sample, which results in promoted martensite transformation. As verified in Figure 8 and Figure 9, martensitic phase transformation is triggered and enhanced by hetero-deformation in local deformation zones. The strain hardening caused by the TRIP effect can counteract the plastic instability and thus inhibit further local deformation. Thus, the TRIP effect can drive Lüders band to an undeformed region. In total, the heterogeneous structure is conducive to the stable propagation of the plastic instability (Lüders band) by changing the stress state distribution inside the sample due to the non-coordinated deformation between the layers, while the TRIP effect is based on its own strain hardening and stress relaxation effects to counteract the plastic instability.

**Figure 13:**
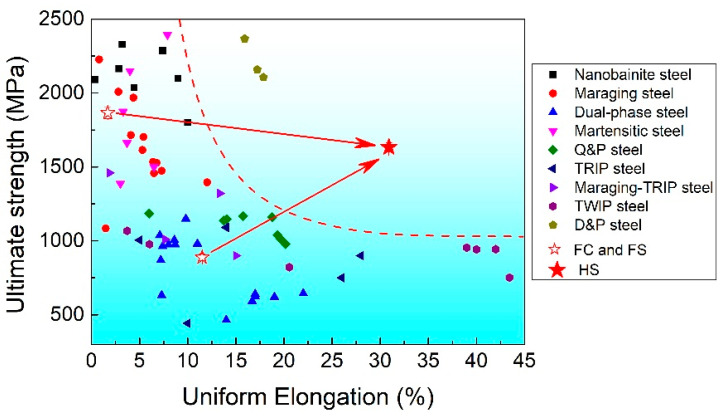
Representative tensile properties of steel. The properties of the studied steels are marked with red stars.

Figure 13 shows the comparison of the tensile properties between the investigated steel and other steel grades. The figure is divided into two regions with red dashed lines. The area on the left represents the strength-ductility trade-off, which is expressed as high strength–low ductility and low strength–high ductility. The area on the right represents the strength–ductility synergy, i.e., high strength–high ductility. It is observed that both FC and FS samples are in the left region, while HS samples clearly achieve strength–ductility synergy. Moreover, the HS samples also show significant mechanical property advantages compared to the existing steel grades. For example, at a strength level of ~1600 MPa, the existing steel grades can only achieve a uniform elongation of ~12.5%, but the HS samples can exceed 30%. This indicates that the present heterogeneous structural steels have excellent strength–ductility synergy. That is, the heterogeneous structure, TRIP effect, and Lüders-like deformation synergistically produce high-strength and high-ductility materials effectively.

## 5. Conclusions

This work shows a heterogeneous “sandwich” structure with equiaxial austenite grains in the central layer and dual-phase nanolamellar on both sides. The heterogeneous structure exhibits excellent tensile properties, combining yield strength of ~1608 MPa and fracture elongation of ~31.2%. By comparing the microstructures of heterogeneous sample and freestanding layers, we draw the following conclusions:

(i) The extra increase in martensite content and hardness was observed in the HS sample. Additionally, the experimentally measured increment is higher than the value calculated by the ROM. This indicates an additional strengthening effect is activated under the influence of heterogeneous structure;
(ii) The presence of the Lüders band and the interfacial influence zone demonstrate that the heterogeneous structure alters the strain distribution and stress state during the tensile deformation;
(iii) The strong constraint between the soft coarse grain core and hard nanolamellar surface layers produced a strain gradient at the interface and increased internal stresses (HDI stress) within the entire sample, which promotes the TRIP effect;
(iv) The Lüders band deformation is stabilized by stress state change within the sample and offset by strain hardening and strain relaxation of the TRIP effect, which enables Lüders bands to propagate steadily under large strains until it covers the whole sample;
(v) Heterogeneous deformation-induced hardening and TRIP effect can provide the extra hardening ability to improve the strength and ductility of the sample. Lüders deformation further improves the total ductility by advancing strain development. Thus, the combined effect of the three is to achieve a high strength–ductility synergy in the present steel.

## Figures and Tables

**Table 1:** Nominal composition of experimental maraging steel (wt.%).

Fe	C	Cr	Ni	Mo	Mn	Cu	V	Nb
Balance	0.4	8	8	4	2	2	0.1	0.1

## Data Availability

Not applicable.
